# Mutations in *COL1A1/A2* and *CREB3L1* are associated with oligodontia in osteogenesis imperfecta

**DOI:** 10.1186/s13023-020-01361-4

**Published:** 2020-03-31

**Authors:** Kristofer Andersson, Barbro Malmgren, Eva Åström, Ann Nordgren, Fulya Taylan, Göran Dahllöf

**Affiliations:** 1 Department of Dental Medicine, Division of Orthodontics and Pediatric Dentistry, Karolinska Institutet, POB 4064, SE-141 04 Huddinge, Sweden; 2 Center for Pediatric Oral Health Research, Stockholm, Sweden; 3 Department of Women’s and Children’s Health, Karolinska Institutet, Stockholm, Sweden; 4 Pediatric Neurology, Astrid Lindgren Children’s Hospital at Karolinska University Hospital, Stockholm, Sweden; 5 Department of Molecular Medicine and Surgery, Center for Molecular Medicine, Karolinska Institutet, Stockholm, Sweden; 6 Department of Clinical Genetics, Karolinska University Hospital, Stockholm, Sweden; 7 Center for Oral Health Services and Research, Mid-Norway, TkMidt, Trondheim, Norway

**Keywords:** Genetics, Hypodontia, Mutation, Tooth agenesis, Tooth development

## Abstract

**Background:**

Osteogenesis imperfecta (OI) is a heterogeneous connective tissue disorder characterized by an increased tendency for fractures throughout life. Autosomal dominant (AD) mutations in *COL1A1* and *COL1A2* are causative in approximately 85% of cases. In recent years, recessive variants in genes involved in collagen processing have been found. Hypodontia (< 6 missing permanent teeth) and oligodontia (≥ 6 missing permanent teeth) have previously been reported in individuals with OI. The aim of the present cross-sectional study was to investigate whether children and adolescents with OI and oligodontia and hypodontia also present with variants in other genes with potential effects on tooth development. The cohort comprised 10 individuals (7.7–19.9 years of age) with known *COL1A1*/*A2* variants who we clinically and radiographically examined and further genetically evaluated by whole-genome sequencing. All study participants were treated at the Astrid Lindgren Children’s Hospital at Karolinska University Hospital, Stockholm (Sweden’s national multidisciplinary pediatric OI team). We evaluated a panel of genes that were associated with nonsyndromic and syndromic hypodontia or oligodontia as well as that had been found to be involved in tooth development in animal models.

**Results:**

We detected a homozygous nonsense variant in *CREB3L1*, p.Tyr428*, c.1284C > A in one boy previously diagnosed with OI type III. *COL1A1* and *COL1A2* were the only two genes among 9 individuals which carried a pathogenic mutation. We found rare variants with unknown significance in several other genes related to tooth development.

**Conclusions:**

Our findings suggest that mutations in *COL1A1*, *COL1A2,* and *CREB3L1* may cause hypodontia and oligodontia in OI. The findings cannot exclude additive effects from other modifying or interacting genes that may contribute to the severity of the expressed phenotype. Larger cohorts and further functional studies are needed.

## Background

Osteogenesis imperfecta (OI; MIM 166200, 166210, 259420, and 166220) is a heterogeneous connective tissue disorder characterized by an increased tendency for fractures throughout life. Other symptoms include growth deficiency, joint laxity, prolonged bleeding tendency, bruises, premature hearing loss, blue sclerae, and dentinogenesis imperfecta (DGI). The reported prevalence of OI is approximately 7 in 100,000 individuals [1]. The disease has traditionally been classified into four main types based on primary clinical findings and pattern of inheritance [2]. Severity ranges from a slightly increased fracture tendency to perinatal lethality. A new OI nomenclature comprising five main types has been proposed [3]; however, the Sillence classification is still the most frequently used.

Autosomal dominant (AD) mutations in the *COL1A1* and *COL1A2* genes are causative in approximately 85% of cases [4]. In addition, mutations in *IFITM5* have been found with an autosomal dominant pattern of inheritance [5]. OI-causing variants often present with unique features, but the interfamilial phenotypic variability can be extensive, despite the same causing mutation [4]. In recent years, recessive variants in genes involved in collagen processing have been found [6]. Among these, Symoens et al. [7] identified a homozygous deletion in cyclic AMP responsive element-binding protein 3-like I (*CREB3L1*), which in one Turkish family caused a severe form of OI. *CREB3L1* encodes a transcription factor which is also known as OASIS (Old Astrocyte Specifically Induced Substance).

Tooth agenesis denotes congenital absence of one or more permanent teeth. Hypodontia is defined by agenesis of fewer than six teeth, while oligodontia is congenital absence of six or more permanent teeth, excluding third molars. Tooth agenesis can occur either as an isolated trait (nonsyndromic) or as part of a syndromic trait (syndromic), which reflects the genetic and phenotypic heterogeneity of the condition [8]. The prevalence of hypodontia in the general population ranges from 6 to 8% in Nordic studies [9–11] with oligodontia occurring in 1–2‰ [11–14]. Mutations in several genes have been associated with nonsyndromic tooth agenesis, among others the *PAX9*, *MSX1, AXIN2*, *EDA*, *EDARADD*, and *EDAR* genes [15–19]. Tooth agenesis has previously been reported in patients with OI [20, 21]. Malmgren et al. [22] found hypodontia in 11%, and oligodontia in 6% of children and adolescents with varying severity of OI. Seventy-five percent of the individuals presenting with oligodontia harbored mutations in *COL1A1* and *COL1A2* that were predicted to cause qualitatively changed protein.

Tooth development is a process under strict genetic control [23–25]. The complex process is regulated by a number of genes including transcription factors, growth factors, signaling molecules and other intra- and extracellular molecules [26].

Phenotypic variability of individuals with mutations in *COL1A1/A2* brings the question whether these individuals have other pathogenic mutations that exclusively cause tooth agenesis. In this study, we investigated 10 OI patients with and without tooth agenesis harboring a *COL1A1/A2* variant in order to identify whether these individuals present with pathogenic mutations in other coding genes that have an important role in tooth development.

## Subjects and methods

This cross-sectional study included 10 children and adolescents (aged 7.7–19.9 years) with OI who had participated in a previous study [22]: 7 presenting with oligodontia; 2 presenting with hypodontia; and a participant from this previous study who had OI type IV but who was newly diagnosed with oligodontia. We evaluated the cohort further genetically. 3 participants were classified as OI type I, 4 as OI type III, and 3 as OI type IV (Table 1).

**Table 1:** Clinical and genetic findings in individuals further investigated with whole-genome sequencing (WGS) (*n* = 10)

Pat No.	OI type	Gender	Missing teeth	DGI	Gene	Mutation, cDNA**	Mutation, protein	Qual. = 1 Quant. = 0***	Heredity* cDNA**	Agenesis in parents
1	IV	M	15, 14, 24, 25, 34, 35, 44, 45	0	*COL1A1*	c.2461G > A	p.(Gly821Ser)	1	de novo mutation	mother, 3 teeth
2	IV	F	15, 14, 24, 25, 34, 35, 44, 45	1	*COL1A2*	c.3106G > C	p.(Gly1036Arg)	1	unknown (adopted)	unknown
3	I	F	15, 14, 24, 25, 35, 44, 45	1	*COL1A2*	c.856G > A	p.(Gly286Ser)	1	de novo mutation	none
4	III	F	15, 14, 24, 25, 34, 35, 45	0	*COL1A1*	c.3118G > A	p.(Gly1040Ser)	1	de novo mutation	none
5	I	M	15, 14, 25, 37, 44, 45, 47	0	*COL1A1*	c.1299 + 1G > A	Splice variant	0	maternal	+
6	III	M	15, 14, 25, 35, 44, 45	0	*CREB3L1*	c.1284C > A	Nonsense	0	de novo mutation	none
7	III	F	15, 14, 24, 25, 35, 45	1	*COL1A1*	c.2075G > C	p.(Gly692Ala)	1	de novo mutation	twin 6 teeth
8	III	F	15, 24, 25, 35, 45	1	*COL1A1*	c.2075G > C	p.(Gly692Ala)	1	de novo mutation	twin 5 teeth
9	I	M	15, 14, 24, 25, 34	1	*COL1A2*	c.3089G > C	p.(Gly1030Ala)	1	de novo mutation	none
10	IV	F	16, 15, 14, 13, 23, 25, 26	0	*COL1A1*	c.4292C > T	p.(Thr1431Ile)	1	de novo mutation	none

* de novo based on phenotype

**cDNA positions *COL1A1*: ENST00000225964 (NM_000088.3); *COL1A2*: ENST00000297268 (NM_000089.3);

*CREB3L1*: ENSG00000157613 (NM_052854.3)

***Qual. = predicted qualitatively changed protein, quant. = predicted quantitatively changed protein

All study participants had been treated at the Astrid Lindgren Children’s Hospital at Karolinska University Hospital, Stockholm (Sweden’s national multidisciplinary pediatric OI team). All children and adolescents underwent a detailed clinical and radiographic evaluation of panoramic radiographs performed by two experienced pediatric dentists (KA and BM) regarding total number of permanent tooth germs and teeth. All included individuals were aged > 7 years to enable evaluation of all permanent teeth. In cases of dental agenesis, a family history was taken. The clinical examination included recording signs of DGI.

Venous blood samples were collected from all individuals. Furthermore, seven family members of patient no.1 accepted to provide their saliva samples for segregation analysis. Genomic DNA was isolated at the Clinical Genetics Unit of Karolinska University Hospital.

Written informed consent for participation and sample collection was obtained from all recruited individuals and/or their legal guardians in accordance with the Declaration of Helsinki. The Swedish regional ethics committee in Stockholm approved the study protocol (Daybook no. 157/99, 2014/254–31, and 2012/2106–31/4).

### Whole-genome sequencing and bioinformatics analysis

Libraries were prepared for sequencing on Illumina HiSeqX (Illumina Inc., San Diego, CA, USA) from the genomic DNA using the Illumina TruSeq DNA PCR-Free kit with a mean insert size of 400 bp. On average, this resulted in approximately 480 million mapped unique sequences with a mean coverage of > 37, i.e., 30x coverage for 80% of reference sequences. We used an in-house pipeline developed by the Science for Life Laboratory, Stockholm, Sweden to map reads to the human reference genome (hg19) and to call variants. Data were aligned to the reference genome using bwa (v0.7.12) [27].

We deduplicated, recalibrated, and indel realigned raw alignments using GATK (v3.3–0-geee94ec) [28]. The quality control information was gathered using Qualimap (v2.2) [29] and single-nucleotide variants (SNVs) and indels were called using the HaplotypeCaller in GATK. Supplementary Table 1 presents general statistics of WGS data to show sequencing quality. We further processed the variants with GenotypeGVCFs, VariantRecalibrator, ApplyRecalibration, VariantFiltration and SelectVariants tools in GATK (v3.7), which were then functionally annotated using Variant Effect Predictor (VEP; version 89) [30] and loaded into a database using GEMINI (v0.20.0) [31]. We followed two approaches. We excluded variants with minor allele frequency of 0.1% and higher in 1000 Genomes Project (1000G), 6500 NHLBI-GO Exome Sequencing Project (ESP), Swedish Genome Project (SweGen) [32] and Exome and Genome Aggregation Consortium (ExAC and gnomAD). Variants located in repetitive sequence regions were excluded. Only non-synonymous variants, frameshift indels, and putative splice site variants were considered for further analysis. We used Combined Annotation Dependent Depletion (CADD) to score the pathogenicity of SNVs [33]. Evolutionary conservation of variants was evaluated with Genomic Evolutionary Rate Profiling (GERP) [34]. The variants were explored in the database using built-in tools in GEMINI, and variants were visualized on Integrated Genome Viewer (IGV) [35].

Structural variants were analyzed using the FindSV pipeline (https://github.com/J35P312/FindSV) which merges calls from CNVnator V0.3.2 [36] and TIDDIT [37]. The structural variants were annotated by VEP and filtered based on the quality flag of the variant. The filtered and annotated variants were then sorted based on a local structural variant frequency database consisting of variants from 1000 healthy individuals from the SweGen project. The reads at breakpoints were visualized in the IGV. The structural variants that fall into intergenic regions as well as intronic deletions and duplications were excluded.

Based on literature reviews, in a systematic manner, we created a panel of genes that are associated with nonsyndromic and syndromic hypodontia or oligodontia as well as of genes involved in tooth development in animal models [8, 38, 39] (Supplementary Table 2). In our downstream analysis of the whole-genome sequencing (WGS) data, we also used this gene panel to investigate both single nucleotide and structural variants in genes that have roles in tooth agenesis and tooth development. Here, we applied a minor allele frequency higher than 1% for the population databases to filter out common single nucleotide variants. In the structural variant analysis, we focused only on breakpoints located in the exons of coding genes that were listed in our gene list (Supplementary Table 2).

### PCR and sanger sequencing

We performed PCR and Sanger sequencing to validate variants of interest and for segregation analysis using standard PCR and Sanger sequencing protocols. Primers are available upon request.

## Results

### Clinical findings and confirmation of *COL1A1/A2* variants

**Fig. 1:**
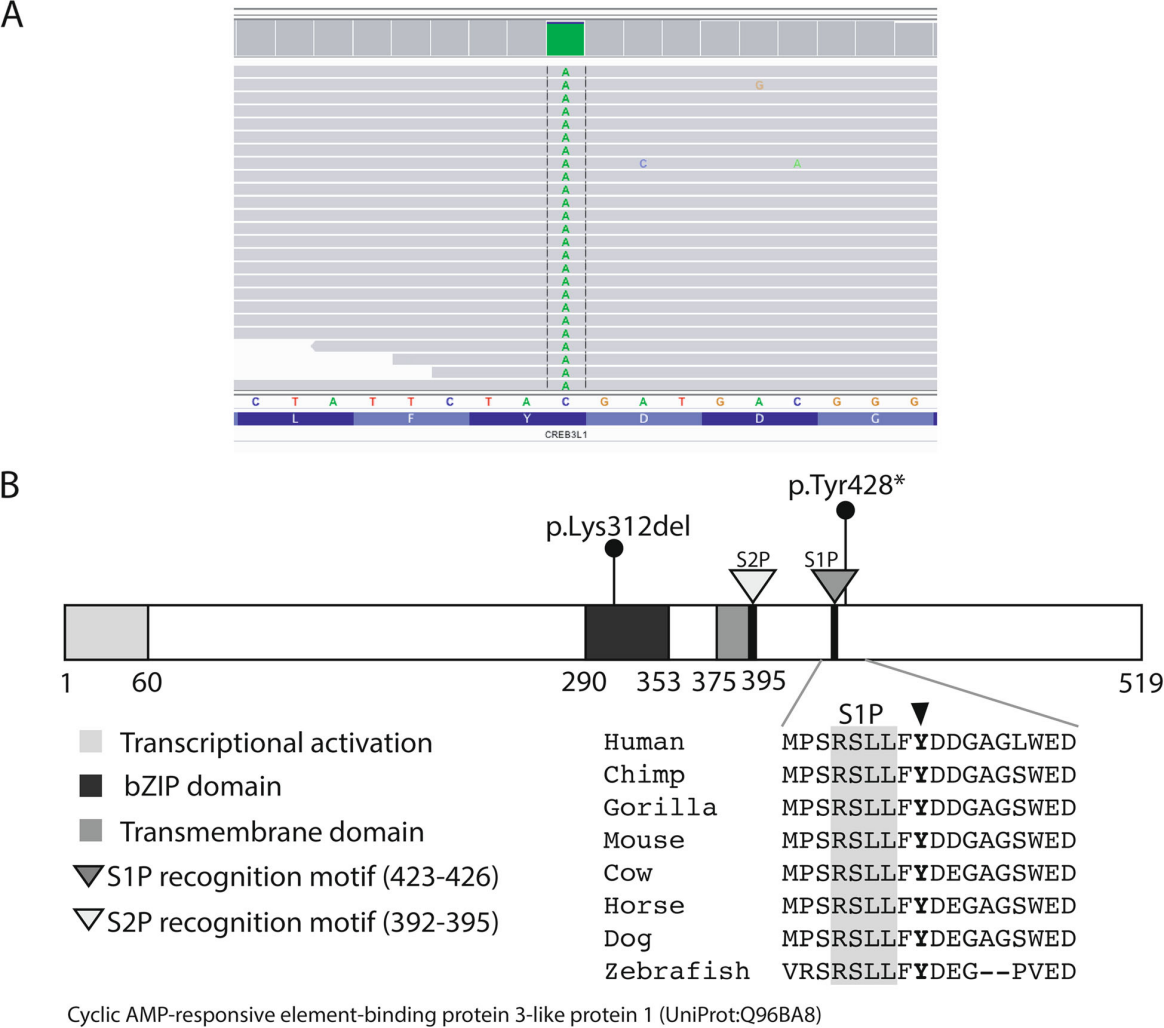
The *CREB3L1* variant visualized on an Integrated Genome Viewer (IGV) and schematic representation of the domain structures of human OASIS. (**a**) The homozygous variant at chr11(hg19): 46341840 C > A in whole genome sequencing data on IGV in patient no 6. The variant is located in *CREB3L1*. It creates a stop codon at position 428 and causes early termination of the protein (p.Tyr428*, c.1284C > A). (**b**) Schematic representation of the domain structures of human OASIS. Different domains are colored in different shades of grey. Mutations identified in this study (p.Tyr428*) and identified by Keller et al. (p.Lys312del) have been shown on the protein. The mutation is located directly after the S1P recognition motif (RSLL), highlighted in grey. Multiple sequence alignment shows evolutionary conservation of Tyr428 across species

Mean age at the most recent evaluation of dental phenotype in the 10 individuals was 12.8 ± 3.7 years (range 7.7–19.9 years). Missing premolars were the most frequent finding, 92% (61/66). The individuals included into this study presented with a heterozygous variant in *COL1A1*, 70% (7/10) and *COL1A2* (3/10). The majority of *COL1A1* and *COL1A2* variants were de novo mutations (Table 1). Whole genome sequencing confirmed pathogenic *COL1A1/A2* mutations in 9 of the 10 individuals. One boy (patient no. 6) previously diagnosed with OI type III had been diagnosed with a splice variant in *COL1A1* (c.3208-6C > T). This variant was assessed as likely benign in this investigation since it is a common single-nucleotide polymorphism (SNP) (gnomAD v2.1 MAF: 0.00068) and we concluded that it was not responsible for the patient’s OI phenotype. However, we identified a homozygous nonsense variant in *CREB3L1*, p.Tyr428*, c.1284C > A (NM_052854.3) (Fig. 1a). At the time of analysis, this variant was not found in any publicly available population database but is recently published [40]. It creates a stop codon, resulting in a premature termination of protein at position 428 instead of 519. The CADD score was very high (36.0) and Tyr428 is evolutionarily conserved across species (Fig. 1b). The mutation was located on the C-terminal of OASIS which is localized in the rough endoplasmic reticulum. Based on the literature, it was assessed as the most likely disease-causing variant, and we discarded the *COL1A1* variant.

**Fig. 2:**
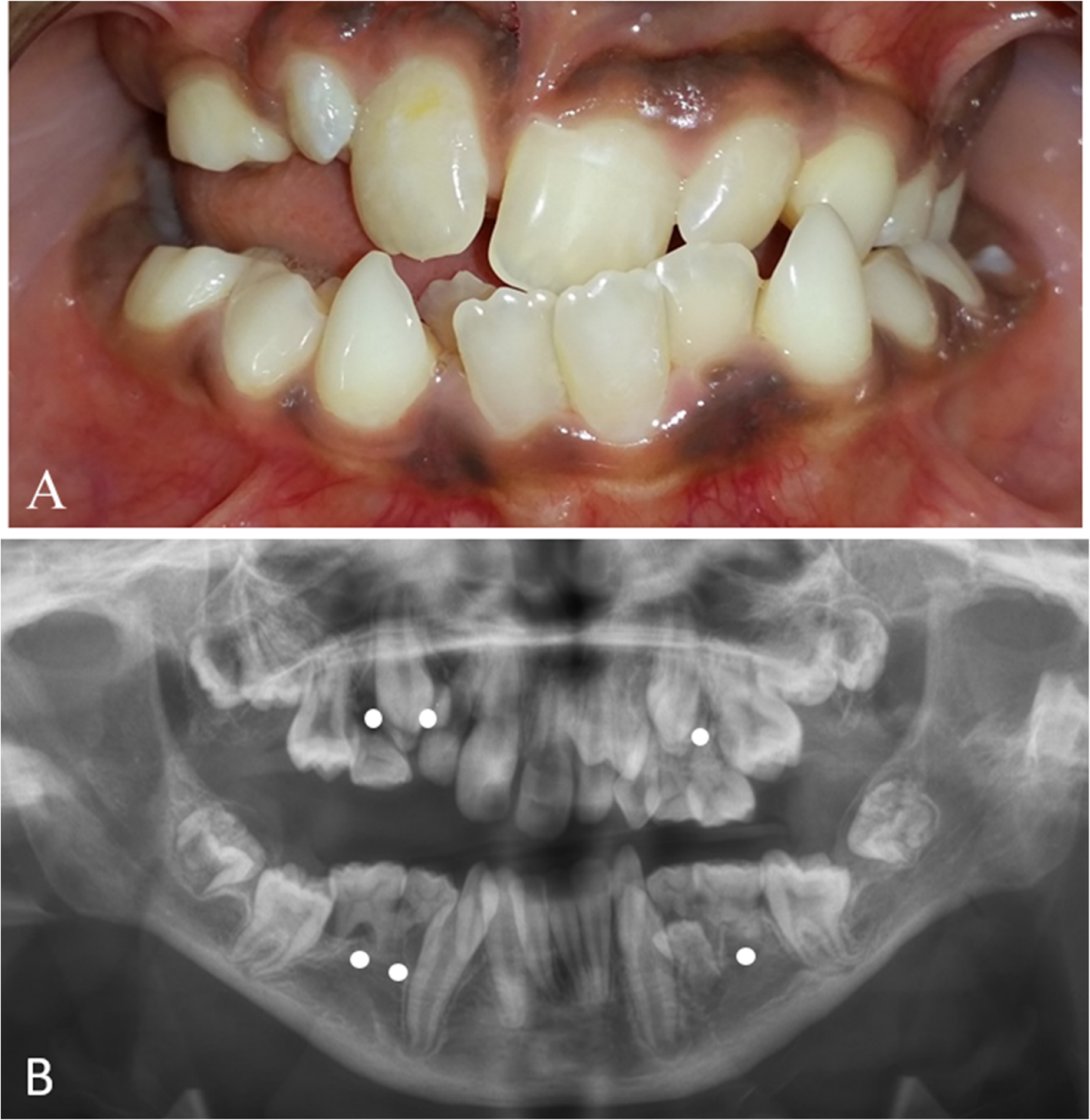
Clinical and radiographic findings in a 12-year-old boy identified with a *CREB3L1* variant. (**a**) A severe malocclusion with crowding and lateral open bite on the right side and cross-bite on the left. No clinical signs of dentinogenesis imperfecta (DGI). (**b**) Panoramic radiograph showing absence of six permanent tooth germs in the premolar regions. The radiograph is difficult to interpret due the extensive crowding. No signs of DGI

The boy harboring the *CREB3L1* variant (patient no. 6) had no family history of OI or tooth agenesis. None of the four siblings were affected by OI or tooth agenesis. At the time of the oral examination, the proband presented with a mixed dentition. Investigation revealed six missing permanent tooth germs, all premolars, and a severe malocclusion including mandibular overjet, unilateral open bite, and bimaxillary crowding (Fig. 2). The boy exhibited neither clinical nor radiographic signs of DGI in the deciduous or permanent dentitions (Table 1). Further details regarding the non-dental findings in the proband have recently been described in detail [40].

### Genomic analyses

#### Rare variants in genes related to tooth development

Variants in genes known to be relevant in tooth development were found in all individuals (Table 2). Excluding *COL1A1* and *COL1A2*, we identified 23 heterozygous variants in 21 genes. The majority (*n* = 21) were SNVs resulting in missense variants in highly conserved positions (Table 2). Of the identified missense variants, 17% (4/23) could be filtered out as mutations in these genes cause other conditions that were not phenotypically expressed by our probands (*BMPER*-diaphanospondylodysostosis with AR inheritance; *GLI3*-mainly developmental disorders with AD inheritance pattern; *ERBB3*-lethal congenital contracture syndrome 2, and *PTCH1*-Gorlin syndrome). These variants were thus deemed unlikely to be the cause of the patients’ symptoms.

**Table 2:** Detected heterozygous variants with high pathogenicity score in highly conserved regions in genes related to tooth development (*n* = 23)

Pat. No.	Chrom^a^	Start	End	Gene	Transcript ID	Aa^**b**^ change	Aa^**b**^ Position/Total length	Impact	GERP	CADD c-score
1	chr4	99,960,545	99,960,546	*METAP1*	ENST00000296411	A/V	121/386	Missense variant	4.94000005722	23.1
1	chr9	98,220,506	98,220,507	*PTCH1*	ENST00000331920	D/Y	986/1447	Missense variant	3.32999992371	26.4
2	chr7	42,005,900	42,005,901	*GLI3*	ENST00000395925	A/T	924/1580	Missense variant	4.84999990463	32.0
2	chr1	208,227,830	208,227,831	*PLXNA2*	ENST00000367033	R/C	931/1894	Missense variant	5.38000011444	32.0
2	chr14	95,582,934	95,582,935	*DICER1*	ENST00000343455	R/H	536/1922	Missense variant	5.11999988556	34.0
3	chr2	17,997,866	17,997,867	*MSGN1*	ENST00000281047	D/Y	28/193	Missense variant	4.71999979019	25.3
3	chr12	99,120,953	99,120,954	*APAF1*	ENST00000339433	W/R	1069/1163	Missense variant	5.25	26.9
3	chr12	56,486,591	56,486,592	*ERBB3*	ENST00000267101	R/W	391/1342	Missense variant	4.23000001907	34.0
5	chr1	120,468,377	120,468,378	*NOTCH2*	ENST00000256646	G/A	1354/2471	Missense variant	5.84000015259	24.9
5	chr1	202,742,409	202,742,414	*KDM5B*	ENST00000367264	LV/X	136–137/1580	Frameshift variant	5.78999996185	None
6	chr6	106,536,187	106,536,188	*PRDM1*	ENST00000369091	A/D	16/789	Missense variant	5.80000019073	21.3
6	chr1	120,496,273	120,496,274	*NOTCH2*	ENST00000256646	N/D	753/2471	Missense variant	5.98999977112	23.3
6	chr3	185,797,885	185,797,886	*ETV5*	ENST00000306376	D/H	124/510	Missense variant	5.32000017166	24.5
6	chr4	20,555,444	20,555,445	*SLIT2*	ENST00000273739	P/R	864/1542	Missense variant	5.11999988556	31.0
6	chr14	35,786,486	35,786,487	*PSMA6*	ENST00000261479	L/R	239/246	Missense variant	5.21000003815	32.0
7, 8	chr3	50,211,278	50,211,279	*SEMA3F*	ENST00000002829	D/Y	56/785	Missense variant	5.25	25.2
9	chr7	55,249,011	55,249,012	*EGFR*	ENST00000275493	D/E	770/1210	Missense variant	5.84999990463	21.7
9	chr7	34,101,629	34,101,630	*BMPER*	ENST00000297161	R/T	350/685	Missense variant	5.17999982834	22.8
9	chr5	31,521,293	31,521,294	*DROSHA*	ENST00000344624	R/G	295/1374	Missense variant	6.17000007629	23.3
10	chr5	102,361,028	102,361,029	*PAM*	ENST00000274392	A/P	796/875	Missense variant	4.86999988556	24.6
10	chr3	48,698,790	48,698,791	*CELSR3*	ENST00000164024	R/H	426/3312	Missense variant	5.82999992371	27.0
10	chr12	56,490,960	56,490,961	*ERBB3*	ENST00000267101	R/W	803/1342	Missense variant	3.68000006676	34.0
10	chr6	36,177,587	36,177,589	*BRPF3*	ENST00000339717	E/^a^	588/935	Stop gained	5.92000007629	42.0

^a^*Chrom* chromosome number, ^b^*Aa* amino acid

After filtering according to our protocol, two missense variants in *NOTCH2* were considered interesting from a biological perspective: one in patient no. 5 (p.(Gly1354Ala), c.4061G > C), and one in patient no. 6 (p.(Asn753Asp), c.2257A > G). According to GERP predictions, both variants occur at highly conserved positions. One frameshift variant was found in *KDM5B*, and a premature stop in *BRPF3* was assessed as a multi-nucleotide polymorphism (MNP). Both genes are important for regulation of epigenetic events.

#### Variants in known genes associated with tooth agenesis and tooth development

We focused on genes (Supplementary Table 2) that were previously reported to cause hypodontia/oligodontia in humans and play a role in tooth development in mouse models [8, 38, 39]. Here, we applied a lenient filter, set the minor allele frequency (MAF) threshold to 1%, and kept rare variants. We identified 26 variants in 26 genes related to tooth development based on the literature. All individuals were carriers of rare variants in genes of interest. Of the variants, 11 were assessed as probably/possibly damaging by PolyPhen-2 prediction and were identified in 70% (7/10) individuals (Tables 3 and 4).

**Table 3:** Detected heterozygous variants in selected genes related to tooth development based on literature (*n* = 26)

Pat. No.	Chrom^a^	Start	End	Gene	Transcript	Codon change	Aa^b^ change	Aa^b^ Position/Total length	Impact	Polyphen pred^c^	GERP	CADD
1	chr17	63,533,621	63,533,622	*AXIN2*	ENST00000307078	aCg/aTg	T/M	511/843	Missense variant	Probably damaging	5.08	24.7
1	chr19	3,527,018	3,527,019	*FZR1*	ENST00000395095	aaC/aaT	N	143/496	Synonymous variant	–	−7.42	9.43
1	chr2	70,683,580	70,683,581	*TGFA*	ENST00000295400	gcG/gcA	A	85/160	Synonymous variant	–	−11.1	18.95
1	chr20	10,393,438	10,393,439	*MKKS*	ENST00000347364	Gca/Tca	A/S	242/570	Missense variant	Probably damaging	5.71	29.3
1	chr9	98,220,506	98,220,507	*PTCH1*	ENST00000331920	Gac/Tac	D/Y	986/1447	Missense variant	Probably damaging	3.32	26.4
1	chrX	44,921,995	44,921,996	*KDM6A*	ENST00000377967			−/1401	Splice region variant		2.96	13.3
2	chr17	46,607,103	46,607,104	*HOXB1*	ENST00000239174	gcC/gcA	A	237/301	Synonymous variant	–	6.94	10.1
2	chr2	189,861,948	189,861,949	*COL3A1*	ENST00000304636			−/1466	Splice region variant	–	2.2	7.63
2	chr2	189,871,109	189,871,110	*COL3A1*	ENST00000304636	Gct/Act	A/T	1045/1466	Missense variant	Possibly damaging	2.8	25.7
2	chr7	42,005,900	42,005,901	*GLI3*	ENST00000395925	Gcc/Acc	A/T	924/1580	Missense variant	Possibly damaging	4.85	32.0
2	chr7	128,851,226	128,851,227	*SMO*	ENST00000475779	gCt/gTt	A/V	65/70	Missense variant	Benign	5.17	25.2
2	chr8	145,741,869	145,741,870	*RECQL4*	ENST00000428558	gaT/gaC	D	211/1208	Synonymous variant	–	2.86	0.01
3	chr5	149,762,683	149,762,684	*TCOF1*	ENST00000451292	agG/agT	R/S	973/1525	Missense variant	Benign	0.150	1.76
4	chr16	68,842,671	68,842,672	*CDH1*	ENST00000261769	gGt/gAt	G/D	203/882	Missense variant	Probably damaging	4.82	23.9
4	chr5	176,636,763	176,636,764	*NSD1*	ENST00000347982	aTg/aCg	M/T	186/2427	Missense variant	Benign	5.50	3.09
4	chr4	5,642,346	5,642,347	*EVC2*	ENST00000310917	aCa/aGa	T/R	375/1228	Missense variant	Possibly damaging	3.33	25.6
4	chr4	111,539,693	111,539,694	*PITX2*	ENST00000306732	Gcc/Acc	A/T	188/324	Missense variant	Benign	3.15	17.28
5	chr4	5,721,083	5,721,084	*EVC*	ENST00000264956	gAc/gGc	D/G	95/992	Missense variant	Possibly damaging	3.42	23.8
6	chr12	115,112,599	115,112,600	*TBX3*	ENST00000257566	gaG/gaC	E/D	380/743	Missense variant	Benign	−5.58	1.22
6	chr5	174,151,756	174,151,757	*MSX2*	ENST00000239243	gAg/gTg	E/V	32/267	Missense variant	Benign	3.23	17.0
7,8	chr3	50,211,278	50,211,279	*SEMA3F*	ENST00000002829	Gac/Tac	D/Y	56/785	Missense variant	Probably damaging	5.25	25.2
7,8	chr3	58,135,715	58,135,716	*FLNB*	ENST00000295956	gaC/gaT	D	2077/2602	Synonymous variant	–	−9.81	9.72
7,8	chr4	5,713,114	5,713,115	*EVC*	ENST00000264956	cGc/cCc	R/P	3/992	Missense variant	Possibly damaging	2.20	25.3
9	chr12	49,446,069	49,446,070	*KMT2D*	ENST00000301067	Cgc/Tgc	R/C	466/5537	Missense variant	Unknown	3.31	18.42
9	chr2	200,246,463	200,246,464	*SATB2*	ENST00000260926	atG/atA	M/I	142/733	Missense variant	Probably damaging	5.76	24.6
10	chrX	13,778,622	13,778,623	*OFD1*	ENST00000340096	Att/Ctt	I/L	682/1012	Missense variant	Benign	−2.97	0.03

^a^*Chrom* chromosome number

^b^*Aa* amino acid

^**c**^*Polyphen pred* Polyphen-2 prediction

**Table 4:** Example of prediction tools used for assessment of pathogenicity of identified rare variants

Pat. No.	Gene	ClinVar significance	Polyphen-2 prediction	Polyphen score	SIFT prediction	SIFT score	Minor allele frequency
1	*AXIN2*	Likely benign	Probably damaging	1.0	Tolerated	0.21	0.003
1	*FZR1*	None		None		None	0.00994087453707
1	*TGFA*	None		None		None	0.000338868180278
1	*MKKS*	Uncertain, pathogenic	Probably damaging	0.952	Deleterious	0.01	0.00972556485506
1	*PTCH1*	None	Probably damaging	0.95	Deleterious	0.0	−1.0
1	*KDM6A*	Benign		None		None	
2	*HOXB1*	None		None		None	−1.0
2	*COL3A1*	Likely benign		None		None	0.0079
2	*COL3A1*	Likely benign, other	Possibly damaging	0.632		None	0.0069
2	*GLI3*	None	Possibly damaging	0.504	Deleterious	0.01	8.98580243216e-05
2	*SMO*	None	Benign	0.017	Deleterious low confidence	0.0	0.002
2	*RECQL4*	None		None		None	0.00532454361055
3	*TCOF1*	Likely benign	Benign	0.012		None	0.003
4	*CDH1*	None	Probably damaging	0.992	Tolerated	0.05	1.50001500015e-05
4	*NSD1*	Uncertain	Benign	0.007	Tolerated low confidence	0.14	0.00049447091612
4	*EVC2*	Uncertain, benign	Possibly damaging	0.876	Deleterious	0.0	0.0089
4	*PITX2*	Likely benign, other	Benign	0.02	Tolerated	0.61	0.00612416864787
5	*EVC*	Likely benign, other	Possibly damaging	0.786	Tolerated	0.1	0.00930071821378
6	*TBX3*	None	Benign	0.004	Tolerated	0.84	0.002
6	*MSX2*	None	Benign	0.005	Tolerated	0.06	0.0002457002457
7,8	*SEMA3F*	None	Probably damaging	1.0	Deleterious	0.02	−1.0
7,8	*FLNB*	Likely benign		None		None	0.0082
7,8	*EVC*	Uncertain	Possibly damaging	0.655	Tolerated low confidence	0.38	0.0
9	*KMT2D*	None	Unknown	0.0		None	0.001
9	*SATB2*	None	Probably damaging	0.987	Tolerated	0.12	2.99733237419e-05
10	*OFD1*	Benign	Benign	0.066	Tolerated	0.49	0.00845140442456

One boy (patient no.1) with OI type IV presented with two missense variants: in *AXIN2* (rs200883019; p.Thr511Met, c.1532C > T) and in *PTCH1 (*p.Asp986Tyr, c.2956G > T). Both of these genes are known to be expressed during tooth development. Segregation analysis showed that the mother, the maternal grandfather, and the maternal aunt were carriers of the *AXIN2* variant and presented with hypodontia.

We could not find any rare, likely pathogenic structural variants that disrupt genes involved in tooth development. Furthermore, no significant differences in distribution of variants between individuals with oligodontia and hypodontia could be seen. Except for the rare pathogenic variants in *COL1A1*, *COL1A2*, and *CREB3L1*, we were unable to identify any other pathogenic variant in a shared gene in the cohort that could explain the phenotype of oligodontia.

## Discussion

In this study, we used WGS in our search to identify potential gene candidates that would explain the OI phenotype with presence of oligodontia. We hypothesized that individuals presenting with these features harbored variants in modifying genes related to *COL1A1/A2* and tooth development. We found rare variants with unknown significance in several other genes related to tooth development.

A splice variant in *COL1A1*, c.3208-6C > T, a variant that was not predicted to markedly affect mRNA splicing, had previously been identified using Sanger sequencing in one of our investigated individuals (patient no. 6), a boy diagnosed with OI type III [1, 22, 41]. Using WGS, we detected a rare homozygous stop gain variant in *CREB3L1* (p.Tyr428*, c.1284C > A). *CREB3L1* encodes a transcription factor which is also known as OASIS (Old Astrocyte Specifically Induced Substance), an ER-stress inducer that has been found to be essential to correct expression of collagen type I by affecting transcription of *COL1A1* and secretion of matrix proteins [42]. It has been shown that ER-stress causes the N-terminal part of OASIS to be transported to the nucleus where it binds to the osteoblast-specific unfolded protein response element (UPRE) regulatory region in the promoter of *COL1A1*. This induces a high level of tissue-specific procollagen type I expression [42].

To our knowledge, this is the first time the dental and maxillofacial phenotype of an individual with a *CREB3L1* variant has been described in detail. Our patient presented with oligodontia and severe malocclusion including a mandibular overjet, unilateral open bite, and bimaxillary crowding. However, no DGI could be detected clinically or radiographically. We previously reported that presence of clinical and radiographic signs of DGI is associated with mutations in *COL1A1* and *COL1A2* that cause a qualitatively changed protein [41]. Based on our findings, we suggest that homozygous loss-of-function mutations of *CREB3L1* may contribute to arrest of tooth development in specific dental developmental areas but is not enough to cause severe signs of DGI. The expression of collagen type I is seen early in tooth development. In the rat, expression has been detected in the dental epithelium and mesenchyme during the bud stage and during the cap and bell stages [43]. The secretion of predentin is initiated during the late bell stage of tooth development. This may indicate that cells are more sensitive to *CREB3L1* during earlier stages of tooth development, where the altered secretion of collagen type I and its interaction with modifier genes may contribute to arrest of tooth development and cause dental agenesis. Lindahl et al. found markedly low levels of *CREB3L1* mRNA in primary human osteoblast-like cell (hOB) (16%) and fibroblasts (21%), but collagen I levels were only reduced I hOBs (5–10%) indicating tissue-specific sensitivity [40]. OI-causing loss-of-function mutations in *CREB3L1* have been described in two consanguineous families in two previous studies [7, 44]. These studies reported a 3-bp in-frame deletion (p.Lys312del, c.934_936delAAG) in exon 7 [44] and a homozygous whole gene deletion [7]. In the study by Keller et al. [44], heterozygous carriers of the variants were mildly affected by OI and homozygosity caused prenatal/perinatal lethal OI similar to that seen in OI type II. Except for reduced skull mineralization, no other craniofacial characteristics of probands presenting with homozygous variants in *CREB3L1* have been described. However, a heterozygous carrier was found to have blue sclerae, but normal teeth [7]. Since complete loss of *CREB3L1* causes prenatal/perinatal lethality as in both of the previous cases, we believe that, since the nonsense mutation is relatively close to the 3′ end of *CREB3L1*, the transcript might escape from nonsense-mediated decay and translate into a partially functioning protein with a missing C-terminal end.

Several signaling pathways are of specific importance in tooth development and expressed during all stages of tooth development; these are the TGFβ, Shh, FGF, Wnt, and Eda (ectodysplasin) pathways. Thus, the search for presence of rare variants involved in these pathways was of specific interest in this study. Another potential candidate was *NOTCH2*, a gene involved in the Notch signaling pathway. Two missense variants in *NOTCH2* were interesting from a biological perspective: one in patient no. 5 (p.(Gly1354Ala), c.4061G > C), and one in patient no. 6 (p.(Asn753Asp), c.2257A > G). Both presented with high GERP and CADD scores. *NOTCH2* is a highly conserved mammalian homologue of the Drosophila Notch gene, which encodes a transmembrane protein important for various cell fate decisions including differentiation, proliferation, and apoptosis during development. It has been shown to be expressed during several stages of tooth development in the mouse [45, 46]. Odontoblasts initiate dentinogenesis during the bell stage. Notch2 expression was not observed in odontoblasts at this stage of development [46]. None of our patients with a rare variant in *NOTCH2* exhibited any clinical or radiographic signs of DGI.

One missense variant was found in *GLI3* (p.Ala924Thr, c.2770G > A) in a girl (patient no. 2) with OI type IV. We also detected a *PTCH1* variant *(*p.Asp986Tyr, c.2956G > T) in a boy (patient no. 1) with OI type IV. Both genes are involved in the Shh pathway. Shh signaling is essential in tooth development and can be detected in the oral epithelium from embryonic day 11.0 (E.11) in mice [47]. However, we assessed the *PTCH1* variant to be a less likely candidate as a mutation in this gene would cause Gorlin syndrome (MIM 601309) with features including multiple nevoid basal-cell epitheliomas, jaw cysts, and bifid ribs [48]. Furthermore, the *GLI3* variant could be discarded as no developmental disturbance was present. The boy (patient no. 1) also presented with a variant in *AXIN2* (p.Thr511Met, c.1532C > T). The proband’s mother, grandfather, and one aunt, all carriers of the variant, were unaffected by OI but presented with hypodontia. Interestingly, none of them could be diagnosed with oligodontia. This finding indicates that the more severe phenotype seen in our proband may be due to additive effects of the variants in *COL1A1* and *AXIN2* and their modifying or interacting genes.

However, it is worth noting that one maternal uncle with a wild type allele of *AXIN2*, reported that he was missing one or two permanent teeth. This finding points to the oligogenic/polygenic characteristics of tooth agenesis and may be explained by Falconer’s polygenic threshold model. The hypothesis is based on the assumption that liability to tooth agenesis is multifactorial and follows a normal distribution (hypodontia is diagnosed in 6–8% of individuals in the general population). Hypodontia presents when a particular threshold is exceeded and is shifted in close relatives of an affected individual leading to an increased susceptibility within the family. Whole-genome sequencing of all individuals would have increased the chance for identification of more loci contributing to the phenotype. *AXIN2* encodes the axis inhibition protein 2, a protein that facilitates β-catenin degradation by forming a destruction complex in the canonical Wnt signaling pathway. A nonsense mutation in *AXIN2* (p.Arg656*) was previously found to cause oligodontia and a predisposition to colorectal cancer in a four-generation Finnish family [49]. Another variant (p.Trp663*, c.1989G > A) was associated with oligodontia, ectodermal dysplasia, and neoplastic syndrome [50]. Several additional mutations, mostly missense variants, have also been associated with nonsyndromic tooth agenesis [15, 51–53]. The *AXIN2* variant in this study, having a medium impact severity, was common in the Finnish population (gnomAD v2.1 Finnish: 1.3%). MAF was also common in Northern European populations: 0.03% in the gnomAD, suggesting that it is a common polymorphism in Nordic populations.

Limitations of this study include the small sample size. Nevertheless, the included number of individuals is high within a cohort of subjects with OI and oligodontia. Interpreting functional impact of missense changes in genes involved in tooth development is challenging and functional assays are necessary to understand how these changes can potentially affect the amount, structure and function of the expressed protein and cause a dental phenotype that is variable between individuals with OI. There is also a need for segregation analysis in the investigated individuals to further decrease the list of potential gene candidates. In our bioinformatics evaluation, we focused on protein-coding sequences. We did not evaluate rare variants in non-coding genome. We found a missense variant in *AXIN2*, but detected no pathogenic variants in *MSX1*, *PAX9*, *EDA*, *EDAR*, or *EDARADD*, genes that previously have been found in nonsyndromic oligodontia. Nevertheless, we found common and rare variants in these genes, most of them located outside of coding regions.

Based on the findings in this study, OI is associated with an increased risk of hypodontia as well as oligodontia. It is important to diagnose agenesis at an early age in order to optimize treatment planning and treatment. We suggest a panoramic radiograph at 7 years of age in children and adolescents with OI. We detected oligodontia in all types of OI, indicating that severe agenesis may also be found in individuals with OI of mild severity. Furthermore, our findings indicate that the possibility of undiagnosed OI should be considered in individuals presenting with oligodontia and hypodontia. Further evaluation regarding presence of medical and dental variables associated with OI may be indicated in such evaluation, and have previously been described by us in detail [54]. If suspicion of OI remains, referral to a pediatrician is indicated.

## Conclusions

In summary, we found several rare missense variants in all investigated probands. No significant differences in distribution of variants between individuals with oligodontia and hypodontia could be seen. Aside from the pathogenic variants in *COL1A1*, *COL1A2* and *CREB3L1*, we were unable to identify any other mutual variant related to collagen type I that could explain the phenotype with osteogenesis imperfecta and oligodontia. Based on our findings, we suggest that the cause of the expressed phenotype is the collagen I related mutation, but that additive effects from rare variants in several other known genes participating in dental development may be important. The findings of this study provide a snapshot of and give further insight into the genetic heterogeneity of hypo/oligodontia in individuals with OI. We stress the importance of further studies, including more individuals with OI and oligodontia and OI without oligodontia to evaluate potential gene candidates further. Finally, we suggest *CREB3L1* as a candidate gene to be included in the genetic investigation of individuals presenting with oligodontia.

## Supplementary information

**Additional file 1: Table S1.** Summary statistics of whole-genome sequencing data.

**Additional file 2: Table S2.** Genes involved in tooth development that were evaluated.

## Data Availability

The datasets generated and analyzed during the current study are available from the corresponding author on reasonable request.

## Abbreviations

AD: Autosomal dominant
CADD: Combined annotation dependent depletion
DGI: Dentinogenesis imperfecta
ER: Endoplasmic reticulum
GERP: Genomic evolutionary rate profiling
MAF: Minor allele frequency
MNP: Multi-nucleotide polymorphism
OASIS: Old astrocyte specifically induced substance
OI: Osteogenesis imperfecta
SNV: Single-nucleotide variants
WGS: Whole-genome sequencing
