# Supplementary material for: Mutations in COL1A1/A2 and CREB3L1 are associated with oligodontia in osteogenesis imperfecta
Source: Orphanet J Rare Dis. 2020 Mar 31;15:80. doi: 10.1186/s13023-020-01361-4 (PMC7110904; doi:10.1186/s13023-020-01361-4)
Supplement: Supplementary file 1 — Additional file 1: Table S1. Summary statistics of whole-genome sequencing data. [file 13023_2020_1361_MOESM1_ESM.docx]

**Supplementary table 1.** Summary statistics of whole-genome sequencing data.

| **Pat. no.** | **Avg. GC** | **Insert Size** | **≥ 30X** | **Coverage** | **% Aligned** | **Change rate** | **Ts/Tv** | **M Variants** | **TiTV ratio (novel)** | **TiTV ratio (known)** | **% Dups** |
| --- | --- | --- | --- | --- | --- | --- | --- | --- | --- | --- | --- |
| 1 | 41% | 394bp | 79.1% | 37.0X | 99.6% | 619 | 1.994 | 5.01 | 1.4 | 2.0 | 14.3% |
| 2 | 42% | 386bp | 85.5% | 39.0X | 99.3% | 616 | 1.997 | 5.03 | 1.5 | 2.0 | 10.7% |
| 3 | 41% | 403bp | 80.8% | 37.0X | 99.3% | 617 | 1.996 | 5.02 | 1.4 | 2.0 | 13.6% |
| 4 | 41% | 400bp | 79.4% | 37.0X | 99.5% | 616 | 1.995 | 5.03 | 1.4 | 2.0 | 10.7% |
| 5 | 41% | 402bp | 80.7% | 38.0X | 99.3% | 620 | 1.998 | 5.00 | 1.4 | 2.0 | 12.3% |
| 6 | 41% | 391bp | 80.2% | 38.0X | 99.3% | 561 | 2.003 | 5.52 | 1.6 | 2.0 | 11.8% |
| 7 | 42% | 397bp | 83.2% | 38.0X | 99.4% | 616 | 1.995 | 5.04 | 1.4 | 2.0 | 16.4% |
| 8 | 41% | 398bp | 85.3% | 39.0X | 99.6% | 616 | 1.994 | 5.03 | 1.4 | 2.0 | 11.5% |
| 9 | 41% | 398bp | 80.9% | 39.0X | 99.3% | 612 | 1.997 | 5.06 | 1.5 | 2.0 | 10.1% |
| 10 | 42% | 402bp | 81.1% | 37.0X | 99.4% | 618 | 2.002 | 5.01 | 1.5 | 2.0 | 11.4% |

**Pat.no.: Patient number, Avg. GC: Average GC content, Insert Size: Median insert size, ≥ 30X: Fraction of genome with at least 30X coverage, Coverage: Median coverage, % Aligned: % mapped reads, Change rate: Change rate, Ts/Tv: Transitions/transversions ratio, M Variants: Number of variants before filter (millions), TiTV ratio (novel): TiTV ratio from variants NOT found in 'hapmap', TiTV ratio (known): TiTV ratio from variants found in 'hapmap', % Dups: MarkDuplicates - Percent duplication*
