# Supplementary material for: Mutations in COL1A1/A2 and CREB3L1 are associated with oligodontia in osteogenesis imperfecta
Source: Orphanet J Rare Dis. 2020 Mar 31;15:80. doi: 10.1186/s13023-020-01361-4 (PMC7110904; doi:10.1186/s13023-020-01361-4)
Supplement: Supplementary file 2 — Additional file 2: Table S2. Genes involved in tooth development that were evaluated. [file 13023_2020_1361_MOESM2_ESM.docx]

**Supplementary table 2.** Genes involved in tooth development that were evaluated.

| #Chromosome/  scaffold name | Gene start (bp) | Gene end (bp) | Gene name | Associated condition | Reference |
| --- | --- | --- | --- | --- | --- |
| chr1 | 75594119 | 75627218 | *LHX8* | Role in tooth development in mouse | Balic and Thesleff (2015) |
| chr1 | 120202421 | 120286838 | *PHGDH* | Hypodontia | Ye and Attaie (2016) |
| chr1 | 209959036 | 209979465 | *IRF6* | Tooth agenesis | Phan et al (2016) |
| chr1 | 236511562 | 236648214 | *EDARADD* | Oligodontia | Ye and Attaie (2016) |
| chr2 | 69240310 | 69476459 | *ANTXR1* | Pseudoanodontia | Ye and Attaie (2016) |
| chr2 | 70674412 | 70781325 | *TGFA* | Tooth agenesis | Phan et al (2016) |
| chr2 | 88747726 | 88752131 | *FOXI3* | Role in tooth development in mouse | Balic and Thesleff (2015) |
| chr2 | 109510927 | 109605828 | *EDAR* | Oligodontia | Ye and Attaie (2016) |
| chr2 | 121493199 | 121750229 | *GLI2* | Involved in tooth development in mouse | Ye and Attaie (2016) |
| chr2 | 172949468 | 172954405 | *DLX1* | Involved in tooth development in mouse | Ye and Attaie (2016) |
| chr2 | 172964167 | 172967628 | *DLX2* | Involved in tooth development in mouse | Ye and Attaie (2016) |
| chr2 | 174771187 | 174830430 | *SP3* | Role in tooth development in mouse | Balic and Thesleff (2015) |
| chr2 | 189839046 | 189877472 | *COL3A1* | Hypodontia | Ye and Attaie (2016) |
| chr2 | 200134223 | 200335989 | *SATB2* | Oligodontia | Phan et al (2016) |
| chr2 | 219745085 | 219764303 | *WNT10A* | Oligodontia | Ye and Attaie (2016) |
| chr3 | 50192478 | 50226508 | *SEMA3F* | Role in tooth development in mouse | Balic and Thesleff (2015) |
| chr3 | 55499743 | 55523973 | *WNT5A* | Role in tooth development in mouse | Balic and Thesleff (2015) |
| chr3 | 57994127 | 58157982 | *FLNB* | Delayed eruption | Ye and Attaie (2016) |
| chr3 | 110788918 | 110994410 | *PVRL3* | Role in tooth development in mouse | Balic and Thesleff (2015) |
| chr3 | 189349205 | 189615068 | *TP63* | Hypodontia | Phan et al (2016) |
| chr4 | 1795034 | 1810599 | *FGFR3* | Hypodontia | Ye and Attaie (2016) |
| chr4 | 4861393 | 4865663 | *MSX1* | Tooth agenesis | Phan et al (2016) |
| chr4 | 5544499 | 5711275 | *EVC2* | Tooth agenesis | Ye and Attaie (2016) |
| chr4 | 5712924 | 5830772 | *EVC* | Tooth agenesis | Ye and Attaie (2016) |
| chr4 | 88529681 | 88538062 | *DSPP* | Role in tooth development in mouse | Balic and Thesleff (2015) |
| chr4 | 107842959 | 108204963 | *DKK2* | Role in tooth development in mouse | Balic and Thesleff (2015) |
| chr4 | 108968701 | 109090112 | *LEF1* | Involved in tooth development in mouse | Ye and Attaie (2016) |
| chr4 | 111538579 | 111563279 | *PITX2* | Oligodontia | Ye and Attaie (2016) |
| chr5 | 44303646 | 44389808 | *FGF10* | Hypodontia | Ye and Attaie (2016) |
| chr5 | 52776239 | 52782964 | *FST* | Involved in tooth development in mouse | Ye and Attaie (2016) |
| chr5 | 141689992 | 141706020 | *SPRY4* | Role in tooth development in mouse | Balic and Thesleff (2015) |
| chr5 | 149340300 | 149373018 | *SLC26A2* | Hypodontia | Ye and Attaie (2016) |
| chr5 | 149737202 | 149779871 | *TCOF1* | Hypodontia | Ye and Attaie (2016) |
| chr5 | 174151536 | 174157896 | *MSX2* | Role in tooth development in mouse | Balic and Thesleff (2015) |
| chr5 | 176560026 | 176727216 | *NSD1* | Premolar missing | Ye and Attaie (2016) |
| chr5 | 178537852 | 178772431 | *ADAMTS2* | Hypodontia | Ye and Attaie (2016) |
| chr6 | 1610681 | 1614127 | *FOXC1* | Oligodontia | Ye and Attaie (2016) |
| chr6 | 45295894 | 45632086 | *RUNX2* | Involved in tooth development in mouse | Ye and Attaie (2016) |
| chr6 | 50786436 | 50815326 | *TFAP2B* | Agenesis | Ye and Attaie (2016) |
| chr6 | 121756838 | 121770873 | *GJA1* | Small teeth | Ye and Attaie (2016) |
| chr7 | 16501106 | 16570205 | *SOSTDC1* | Role in tooth development in mouse | Balic and Thesleff (2015) |
| chr7 | 19060614 | 19157295 | *TWIST1* | Hypodontia | Phan et al (2016) |
| chr7 | 42000548 | 42277469 | *GLI3* | Role in tooth development in mouse | Balic and Thesleff (2015) |
| chr7 | 94023873 | 94060544 | *COL1A2* | Hypodontia | Ye and Attaie (2016) |
| chr7 | 128828713 | 128853386 | *SMO* | Role in tooth development in mouse | Balic and Thesleff (2015) |
| chr7 | 155592680 | 155604967 | *SHH* | Delayed eruption | Ye and Attaie (2016) |
| chr8 | 16849678 | 16859690 | *FGF20* | Role in tooth development in mouse | Balic and Thesleff (2015) |
| chr8 | 38268656 | 38326352 | *FGFR1* | Oligodontia | Phan et al (2016) |
| chr8 | 72109668 | 72274467 | *EYA1* | Dental phenotype | Ye and Attaie (2016) |
| chr8 | 99956631 | 99964332 | *OSR2* | Role in tooth development in mouse | Balic and Thesleff (2015) |
| chr8 | 102504660 | 102681954 | *GRHL2* | Hypodontia | Ye and Attaie (2016) |
| chr8 | 145736667 | 145743229 | *RECQL4* | Hypodontia | Ye and Attaie (2016) |
| chr9 | 89559279 | 89562104 | *GAS1* | Involved in tooth development in mouse | Ye and Attaie (2016) |
| chr9 | 98205262 | 98279339 | *PTCH1* | Hypodontia | Phan et al (2016) |
| chr9 | 124964856 | 124991905 | *LHX6* | Role in tooth development in mouse | Balic and Thesleff (2015) |
| chr9 | 132427920 | 132484875 | *PRRX2* | Involved in tooth development in mouse | Ye and Attaie (2016) |
| chr10 | 54074056 | 54077802 | *DKK1* | Involved in tooth development in mouse | Ye and Attaie (2016) |
| chr10 | 88516407 | 88692595 | *BMPR1A* | Involved in tooth development in mouse | Ye and Attaie (2016) |
| chr10 | 101948055 | 101989376 | *CHUK* | Involved in tooth development in mouse | Ye and Attaie (2016) |
| chr10 | 103529899 | 103535854 | *FGF8* | Involved in tooth development in mouse | Ye and Attaie (2016) |
| chr10 | 123237848 | 123357972 | *FGFR2* | Hypodontia | Ye and Attaie (2016) |
| chr11 | 69587797 | 69590171 | *FGF4* | Role in tooth development in mouse | Balic and Thesleff (2015) |
| chr11 | 69624992 | 69633792 | *FGF3* | Role in tooth development in mouse | Balic and Thesleff (2015) |
| chr11 | 119494120 | 119599794 | *PVRL1* | Oligodontia | Ye and Attaie (2016) |
| chr12 | 49359123 | 49365546 | *WNT10B* | Role in tooth development in mouse | Balic and Thesleff (2015) |
| chr12 | 49412758 | 49453557 | *KMT2D* | Hypodontia | Phan et al (2016) |
| chr12 | 57853918 | 57866045 | *GLI1* | Role in tooth development in mouse | Balic and Thesleff (2015) |
| chr12 | 71833550 | 71980090 | *LGR5* | Role in tooth development in mouse | Balic and Thesleff (2015) |
| chr12 | 115108059 | 115121969 | *TBX3* | Hypodontia | Ye and Attaie (2016) |
| chr13 | 22245522 | 22278637 | *FGF9* | Role in tooth development in mouse | Balic and Thesleff (2015) |
| chr13 | 80910111 | 80915086 | *SPRY2* | Role in tooth development in mouse | Balic and Thesleff (2015) |
| chr14 | 37126773 | 37148920 | *PAX9* | Hypodontia | Phan et al (2016) |
| chr14 | 54416454 | 54425479 | *BMP4* | Role in tooth development in mouse | Balic and Thesleff (2015) |
| chr14 | 76424442 | 76449334 | *TGFB3* | Hypodontia | Phan et al (2016) |
| chr15 | 43235095 | 43398311 | *UBR1* | Oligodontia | Ye and Attaie (2016) |
| chr16 | 3775055 | 3930727 | *CREBBP* | Hypodontia | Ye and Attaie (2016) |
| chr16 | 68771128 | 68869451 | *CDH1* | Tooth agenesis | Phan et al (2016) |
| chr17 | 45922279 | 45933240 | *SP6* | Role in tooth development in mouse | Balic and Thesleff (2015) |
| chr17 | 46605888 | 46608359 | *HOXB1* | Missing teeth | Ye and Attaie (2016) |
| chr17 | 48260650 | 48278993 | *COL1A1* | Hypodontia | Ye and Attaie (2016) |
| chr17 | 59477257 | 59486827 | *TBX2* | Role in tooth development in mouse | Balic and Thesleff (2015) |
| chr17 | 63524681 | 63557765 | *AXIN2* | Tooth agenesis | Phan et al (2016) |
| chr18 | 45357922 | 45457515 | *SMAD2* | Involved in tooth development in mouse | Ye and Attaie (2016) |
| chr18 | 48494410 | 48611415 | *SMAD4* | Role in tooth development in mouse | Balic and Thesleff (2015) |
| chr19 | 571297 | 583493 | *BSG* | Role in tooth development in mouse | Balic and Thesleff (2015) |
| chr19 | 917287 | 921015 | *KISS1R* | Tooth agenesis | Phan et al (2016) |
| chr19 | 3506271 | 3538328 | *FZR1* | Tooth agenesis | Phan et al (2016) |
| chr19 | 12907634 | 12912694 | *PRDX2* | Involved in tooth development in mouse | Ye and Attaie (2016) |
| chr20 | 4101627 | 4168394 | *SMOX* | Involved in tooth development in mouse | Ye and Attaie (2016) |
| chr20 | 6748311 | 6760927 | *BMP2* | Role in tooth development in mouse | Balic and Thesleff (2015) |
| chr20 | 10381657 | 10414870 | *MKKS* | Hypodontia | Ye and Attaie (2016) |
| chr20 | 10618332 | 10654694 | *JAG1* | Oligodontia | Ye and Attaie (2016) |
| chr20 | 10625847 | 10627014 | *JAG1* | Oligodontia | Ye and Attaie (2016) |
| chr20 | 48599536 | 48605423 | *SNAI1* | Role in tooth development in mouse | Balic and Thesleff (2015) |
| chr20 | 55743804 | 55841685 | *BMP7* | Role in tooth development in mouse | Balic and Thesleff (2015) |
| chr22 | 19744226 | 19771116 | *TBX1* | Tooth agenesis | Phan et al (2016) |
| chrX | 13752832 | 13787480 | *OFD1* | Oligodontia | Phan et al (2016) |
| chrX | 20168029 | 20285523 | *RPS6KA3* | Hypodontia | Ye and Attaie (2016) |
| chrX | 39909068 | 40036582 | *BCOR* | Hypodontia/Oligodontia | Phan et al (2016) |
| chrX | 44732757 | 44971847 | *KDM6A* | Missing incisors | Ye and Attaie (2016) |
| chrX | 48367350 | 48379202 | *PORCN* | Hypoplastic teeth | Ye and Attaie (2016) |
| chrX | 68835911 | 69259319 | *EDA* | Oligodontia | Ye and Attaie (2016) |
| chrX | 79270255 | 79287268 | *TBX22* | Hypodontia | Phan et al (2016) |
| chrX | 153769414 | 153796782 | *IKBKG* | Agenesis | Ye and Attaie (2016) |
